# A Rare Case of Partial Volvulus of the Gall Bladder Detected Preoperatively

**DOI:** 10.7759/cureus.19950

**Published:** 2021-11-27

**Authors:** Anadi S Tasa, Sandeep Dey, Dhrubajit Gogoi, Vikramjit Baruah

**Affiliations:** 1 Surgery, Jorhat Medical College and Hospital, Jorhat, IND; 2 Anaesthesiology, Jorhat Medical College and Hospital, Jorhat, IND

**Keywords:** open cholecystectomy, volvulus reduction, general anesthesia, mitral regurgitation, uterine fibroid, gall bladder volvulus

## Abstract

A middle-aged woman with uterine fibroids presented with menorrhagia and diffuse abdominal pain. The patient was anemic, afebrile, anicteric, and had a palpable tender gall bladder. Initial whole abdomen ultrasonography was inconclusive, and computed tomography of her abdomen revealed partial volvulus of the gall bladder. Following optimization, the patient had undergone open cholecystectomy under general anesthesia. Intraoperatively, the gall bladder was distended, with edematous walls and rotated clockwise (270°) along the long axis to the cystic duct. We noted no gangrenous changes, and we performed detorsion of the gall bladder and cholecystectomy. Volvulus of the gall bladder can be associated with high mortality and morbidity. Good clinical examination, a high degree of suspicion given the presentation of the case, proper investigation, and timely management prevented poor outcomes in our case.

## Introduction

Worldwide, 400 cases of volvulus of gall bladder (GB) have been reported as of 2015 [1], with an estimated incidence of one of 365,000 cases of cholelithiasis [2-6]. Volvulus GB is mainly seen among older women with a median age of 77 years [7]. Most cases are diagnosed intraoperatively [8] and have an associated mortality rate of 6% [9]. Preoperative diagnosis is complex, and the patient often presents with an acute abdomen [10]. Owing to the pathophysiology of the disease (long or wide mesentery), the anatomical location of the volvulus GB can vary, and it can mimic as acute appendicitis or ischemic bowel [11,12]. We report the case of a patient with vague complaints of diffuse abdominal pain with a palpable lump in the right hypochondrium, which was volvulus GB, on further evaluation.

## Case presentation

**Figure 1:**
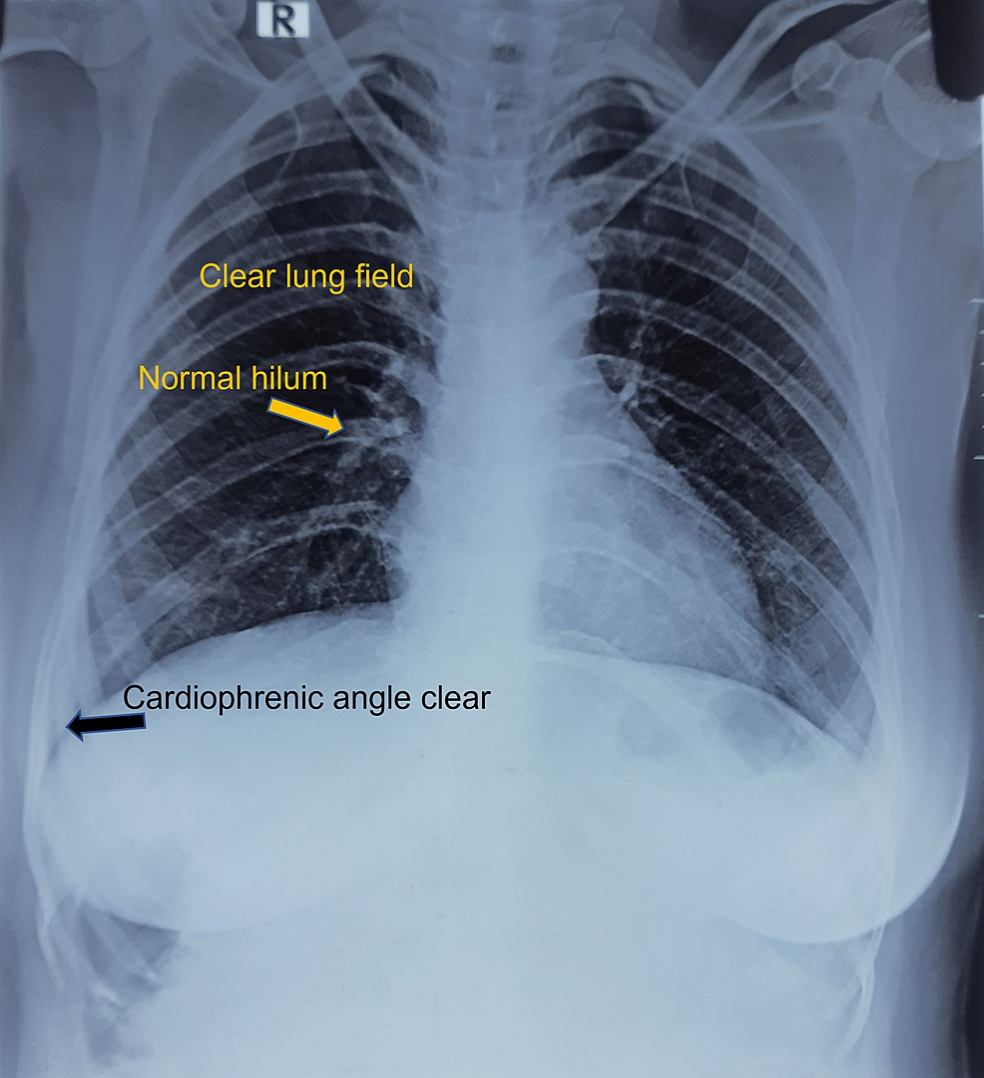
Chest X-ray (PA view) showing clear lung fields PA: posteroanterior.

A 50-year-old woman (47 kg) presented with weakness and abdominal pain lasting around two weeks. The pain was gradual in onset, diffuse in nature, was associated with occasional nausea and vomiting. It was not relieved with sleep or food, but only with medication. There was no history of fever, chill, and rigor, burning micturition, yellowish discolouration of the eyes, or urine. The pain abdomen was associated with menorrhagia, lasting for the same duration. She has a similar history of menorrhagia in the past and was diagnosed with uterine fibroids. She had no history of jaundice, tuberculosis. Bladder and bowel habits were regular. There were no significant symptoms suggestive of any systemic disease. On examination, the patient was conscious, cooperative, afebrile, pale, anicteric, and hemodynamically stable. On examination, there was diffuse tenderness on superficial palpation. A lump was palpable in the right hypochondrium, which was tender, intra-abdominal, and moving up and down with respiration. The surface of the lump was smooth, lower margin, medial and lateral margins were palpable, but the upper margin was passing deep to the costal margin. The liver and spleen were not palpable. No other mass was palpable. External genitalia was normal. Per rectal, per vaginal and systemic examination, was normal. On examination, the patient was afebrile, hemodynamically stable, and had a palpable tender GB. Laboratory tests showed severe anemia (hemoglobin, 6.3 g/dL) with unremarkable complete blood count (8200/mm3). Results from liver function test, serum amylase and lipase, random blood glucose, electrocardiogram (ECG), and chest X-ray findings (Figure 1) were within reference ranges. Her rapid antigen test and TrueNat (Molbio Diagnostics Pvt. Ltd., Bangalore, India) chip-based real-time micro-polymerase chain reaction results for beta coronavirus and severe acute respiratory syndrome coronavirus 2 were negative.

**Figure 2:**
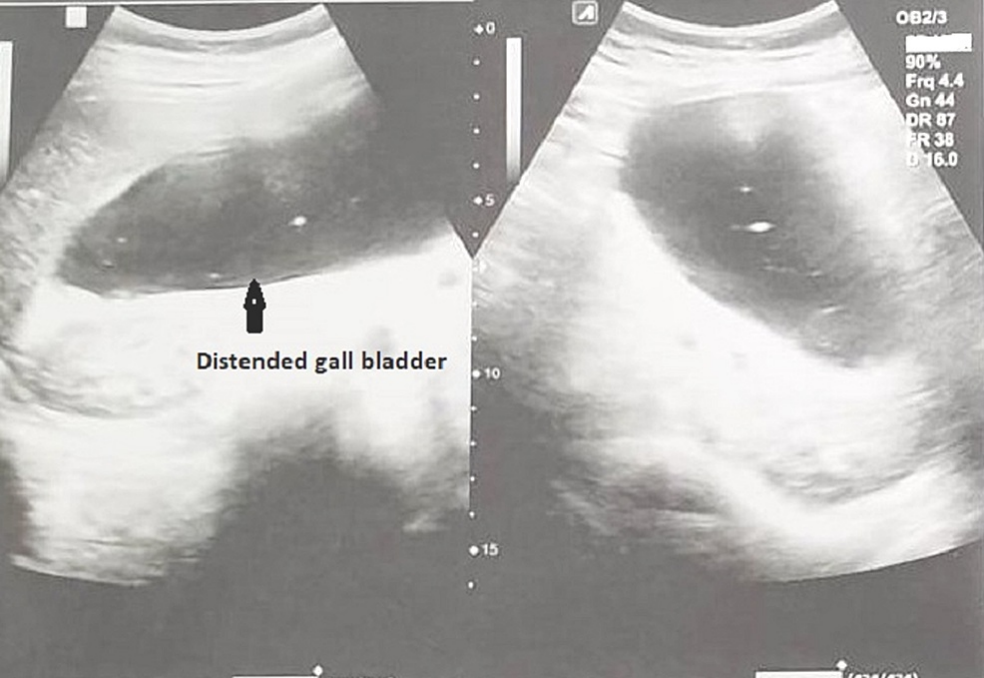
Abdominal ultrasonography showing distended gallbladder

Abdominal ultrasonography revealed a grossly distended GB with suspicious obstruction at the neck. Common bile ducts and biliary ducts (intrahepatic and extrahepatic) were standard calibers with no evidence of sludge or calculi (Figure 2).

**Figure 3:**
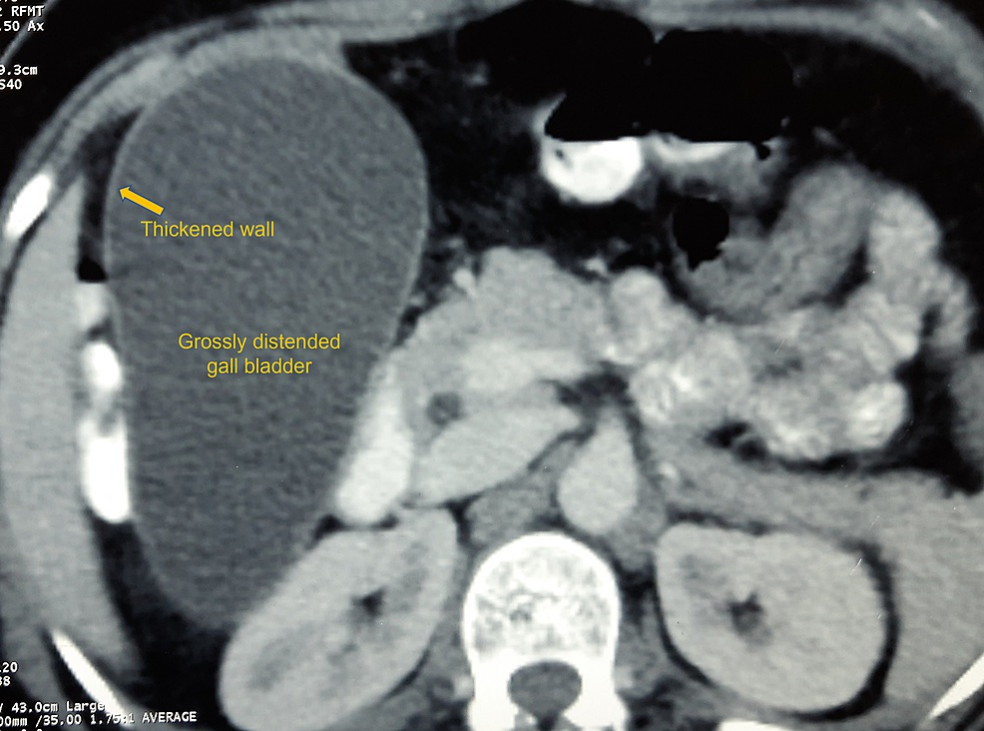
Abdominal computed tomography scan

Since the patient's abdomen was diffusely tender and not associated with fever or jaundice, a diagnosis other than cholelithiasis/cholecystitis was sought and evaluated. A computed tomography (CT) scan of the abdomen revealed a kink at the GB neck and cystic duct junction, with posterolaterally and inferiorly pulled common hepatic duct. The GB was significantly distended and elongated, suggestive of possible partial volvulus of GB. There was minimal sludge in the GB lumen with no biliary obstruction. There was evidence of intestinal ascariasis. We noted a bulky uterus with multiple intramural myomas (Figure 3).

We diagnosed her with uterine fibroid with partial volvulus of the GB and planned an open cholecystectomy under general anesthesia. During her pre-anesthetic check-up, we noted the patient looked ill, pale, anicteric, and she was afebrile, tachycardic (pulse rate: 100 to 110/beats per minute) with blood pressure in the reference range. Her physical status was unremarkable before this episode of abdominal pain, with standard exercise tolerance and no history of shortness of breath, orthopnea, paroxysmal nocturnal dyspnea, chest pain (present or past), and palpitation. She had no significant history of tuberculosis, jaundice, or sore throat in the past. Breathing sounds on auscultation were healthy. There was no evidence of crepitation (fine or coarse). The first heart sound was soft, the second heart sound was typical, and we noted no murmur or additional sounds. Examination of the airway revealed Mallampati Grade I and Cormack-Lehane Grade I airway. Other findings were within reference limits.

Two-dimensional echocardiography revealed mild mitral regurgitation (MR) and healthy left ventricular (LV) function. There was no evidence of left atrial or LV hypertrophy with an LV ejection fraction of 73%.

Since the patient's condition did not call for emergency surgery, nor was there any evidence of volume overload or pulmonary congestion secondary to MR, we decided to optimize her before surgery. Three units of packed cell transfusion over three days were given with diuretic coverage, as necessary, to prevent pulmonary congestion. Deworming and mast cell stabilizers were given as there was evidence of intestinal ascariasis on the CT scan abdomen. She received a broad-spectrum intravenous antibiotic. On the night before surgery, she received a mild anxiolytic (alprazolam 0.25 mg). We planned for elective open cholecystectomy via a right subcostal incision under general anesthesia.

The anesthesia team aimed to maintain preload and avoid bradycardia, hypoxia, hypercarbia, acidosis, and myocardial depression. Standard monitors were attached, including noninvasive blood pressure, pulse rate, oxygen saturation, ECG, and temperature probe. The patient was premedicated with injections of glycopyrrolate (0.01 mg/kg), palonosetron (75 mg), tramadol (1.5 mg/kg), and ketorolac (30 mg, intramuscular). We avoided using sedative premedication, which could lead to hypercarbia or increased pulmonary vascular resistance. A nitroglycerine infusion at 5 µg/minute was started via pump to improve forward LV stroke volume and decrease the regurgitant fraction. The patient was pre-oxygenated with 100% oxygen for three minutes and induced with thiopentone (4 mg/kg injection). Atracurium (0.5 mg/kg) bolus injection was used as a muscle relaxant, and then the patient was intubated. We attached a capnography probe and set mechanical ventilation at 12 breaths/minute to allow sufficient time between breathes for venous return. Anesthesia was maintained using isoflurane inhalation, oxygen, and nitrous oxide, with oxygen maintained at 40% using an oxygen analyzer and atracurium at the titrated dose. As MR patients are sensitive to small changes in intravascular volume status, we monitored central venous pressure to maintain it between 8 cm and 12 cm of water. Also, regular arterial blood gas monitoring was done to avoid hypoxia, hypercarbia, and acidosis that could jeopardize myocardial function. A transdermal ketorolac patch was attached for postoperative analgesia.

**Figure 4:**
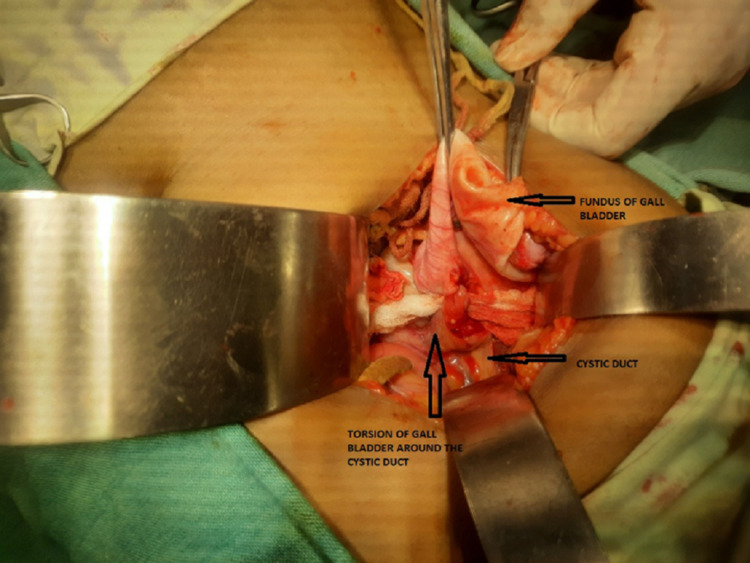
Volvulus of gall bladder showing long mesentery with torsion of the gall bladder around the cystic duct and cystic artery

**Figure 5:**
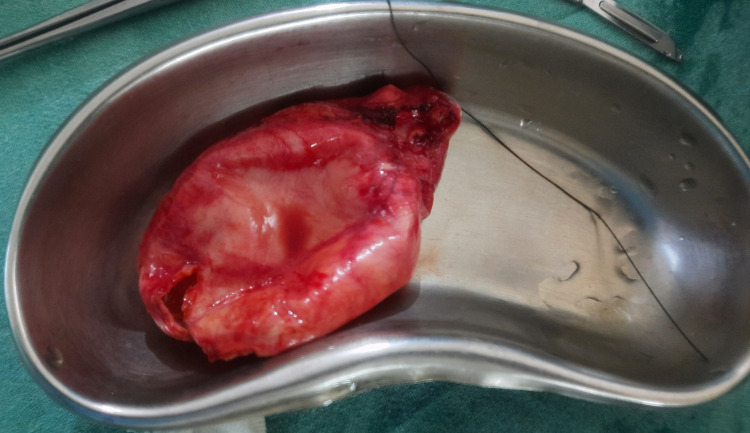
Grossly distended, edematous gall bladder with no gangrenous changes

At surgery, we noted her GB was grossly distended, edematous, rotated clockwise (270°) around the cystic artery and cystic duct, and without any gangrenous changes (Figure 4, Figure 5). Fortunately, the GB was not perforated by this time. As it was an acute disease without gross contamination of the abdominal cavity, we performed a cholecystectomy through Kocher's incision. The volvulus was reduced, GB was removed, and drains were placed in the right subhepatic space.

After complete reversal of the neuromuscular blockade, the patient was extubated. Oxygenation was continued in the postoperative care unit, and when the patient had a modified Aldrete score of nine, she was transferred to the postoperative ward. The postoperative period was uneventful. The patient had complete resolution of pain on a postoperative day one, and she was started a regular soft diet. The histopathology reports suggest cholecystitis with areas of erosion, fibrosis, and focal calcification without acute gangrenous changes. The patient was discharged on postoperative day three. At her two-week postoperative follow-up examination, the patient was doing remarkably well. A consulting gynecologist planned to treat the uterine fibroids at a later date surgically.

## Discussion

According to Dayananda et al., Wendel first described the volvulus of the GB in 1898 as a floating GB [13]. Predominantly seen in women (1:3 male-to-female ratio), 85% of the cases occur in patients aged 60 to 80 years [7,14]. Rarely, children as young as two years may be affected [15].

Volvulus of the GB is characterized by clockwise or counter-clockwise torsion of the GB around the longitudinal axis of the cystic duct and cystic artery. The presence of an abnormally long mesentery from the liver bed to the GB, abnormal vascular pedicle to GB, the peristaltic activity of the surrounding bowel, or spinal deformities predisposes a patient to GB twist. Loss of visceral fat with aging contributes to a higher incidence of GB volvulus in older patients [6,16].

Volvulus GB is a diagnostic challenge and needs a high degree of clinical suspicion. Lau et al. in 1982 described the "Triad of Triads" to help identify the features of GB volvulus [17,18]. Even with advances in radiological diagnosis, only five cases of GB volvulus have been diagnosed preoperatively to date [13-15]. Emergency detorsion and cholecystectomy (laparoscopic or open) is the treatment of choice. Delay in treatment can lead to biliary peritonitis from GB rupture and sequelae [19]. Ours is one of the rare cases of GB volvulus diagnosed preoperatively. The patient initially presented with generalized weakness and diffuse, dull, aching abdominal pain with a history of menorrhagia with GB volvulus. Also, there were no signs or symptoms of acute abdomen or cholecystitis (i.e., abdominal colic, fever, jaundice, or vomiting). Our case only fulfilled five of the nine features described by Lau et al. [17,18], with the absence of spinal deformity, right upper quadrant pain, early emesis, and pulse-temperature discrepancy prevented from fulfilling the "Triad of Triads." The diagnosis was guided by clinical examination by a palpable mass in the right hypochondrium on deep abdominal palpation. Timely evaluation and management led to good patient outcomes and home discharge, rather than GB perforation, biliary peritonitis, or a prolonged hospital stay.

## Conclusions

GB volvulus is among the rarer acute abdomen cases attributed to GB pathology, described mainly among older patients. Given the rarity of preoperative diagnosis even with radiological advancement, this case report highlights the importance of clinical examination of all cases of pain abdomen. Even though her symptoms were suggestive of a gynecological cause, abdominal palpation revealed a palpable mass, subsequently diagnosed as volvulus GB. Timely intervention averted the natural course of the disease and patient morbidity.
